# Pain with neuropathic characteristics after surgically treated lower limb fractures: Cost analysis and pain medication use

**DOI:** 10.1177/20494637231179809

**Published:** 2023-06-15

**Authors:** May Ee Png, Matthew L Costa, Stavros Petrou, Juul Achten, Ruth Knight, Julie Bruce, David J Keene

**Affiliations:** 1 Nuffield Department of Primary Care Health Sciences, 6396 University of Oxford, Oxford, UK; 2 Oxford Trauma and Emergency Care, Kadoorie Research Centre, Nuffield Department of Orthopaedic, Rheumatology and Musculoskeletal Sciences, 6396 University of Oxford, Oxford, UK; 3 Centre for Statistics in Medicine, Oxford Clinical Trials Research Unit, Nuffield Department of Orthopaedics, Rheumatology and Musculoskeletal Sciences, 6396 University of Oxford, Oxford, UK; 4 Warwick Clinical Trials Unit, Warwick Medical School, 2707 University of Warwick, Coventry, UK; 5 University Hospital Coventry and Warwickshire NHS Trust, Coventry, UK; 6 Faculty of Health and Life Sciences, 3286 University of Exeter, Exeter, UK

**Keywords:** Pain, postoperative, musculoskeletal pain, neuropathic pain, injury, orthopaedic surgery, financial cost, medication

## Abstract

**Introduction:**

Neuropathic pain is prevalent among people after lower limb fracture surgery and is associated with lower health-related quality of life and greater disability. This study estimates the financial cost and pain medication use associated with neuropathic pain in this group.

**Methods:**

A secondary analysis using pain data collected over six postoperative months from participants randomised in the Wound Healing in Surgery for Trauma (WHiST) trial. Pain states were classified as pain-free, chronic non-neuropathic pain (NNP) or chronic neuropathic pain (NP). Cost associated with each pain state from a UK National Health Service (NHS) and personal social services (PSS) perspective were estimated by multivariate models based on multiple imputed data. Pain medication usage was analysed by pain state.

**Results:**

A total of 934 participants who provided either 3- or 6-months pain data were included. Compared to participants with NP, those with NNP (adjusted mean difference -£730, *p* = 0.38, 95% CI −2368 to 908) or were pain-free (adjusted mean difference -£716, *p* = 0.53, 95% CI −2929 to 1497) had lower costs from the NHS and PSS perspective in the first three postoperative months. Over the first three postoperative months, almost a third of participants with NP were prescribed opioids and 8% were prescribed NP medications. Similar trends were observed by 6 months postoperatively.

**Conclusion:**

This study found healthcare costs were higher amongst those with chronic NP compared to those who were pain-free or had chronic NNP. Opioids, rather than neuropathic pain medications, were commonly prescribed for NP over the first six postoperative months, contrary to clinical guidelines.

## Introduction

Neuropathic pain is a type of chronic pain defined as ‘pain caused by a lesion or disease of the somatosensory nervous system’ according to the International Association for the Study of Pain (IASP).^1^ Prevalence estimates vary depending on sampling methodology, but approximately 7–8% of adults in the general population report chronic pain with neuropathic characteristics.^2^ There are many causes of neuropathic pain, including traumatic injury of peripheral nerves. Surgical management of a fracture can result in new neuropathic symptoms or exacerbate existing neuropathic pain. The symptoms of neuropathic pain can develop within days of the fracture or take several weeks or months to manifest.^3^

Neuropathic pain substantially reduces health-related quality of life^4^ and people with neuropathic pain consume a substantial amount of healthcare resources. Estimates of direct medical costs attributable to neuropathic pain are around €2951 per patient per year, whilst direct non-medical costs have been estimated at around €1242 per patient per year and indirect costs (i.e. the value of time off work or absenteeism) at around €5492 per patient per year (in 2012 prices).^5^ However, these estimates pertain to all causes of neuropathic pain and not specifically neuropathic pain in people sustaining fractures as a result of major trauma.

Our UK study^6^ found that chronic neuropathic pain was prevalent (30%) amongst people with surgically managed lower limb fractures and was associated with lower health-related quality of life (EQ-5D utility −0.15 (95% CI −0.19 to −0.11); *p* < .001) and greater disability (Disability Rating Index (DRI) adjusted mean difference 11.49 (95% CI 7.84 to 15.14; *p* < .001) over 6 months postoperatively compared to those with chronic non-neuropathic pain or who were pain-free. In the United Kingdom, the National Institute for Health and Care Excellence (NICE) guidelines on chronic pain^7^ and neuropathic pain^8^ make specific recommendations.^9^ In this study, we explore the economic implications of people with chronic neuropathic pain and report pain medication usage for people sustaining lower limb fractures during major trauma. We examine cost and pain medication use over time, in those who reporting pain data at three and 6 months after lower limb surgery for traumatic injury.

## Methods

### Overview of WHiST trial

WHiST was a multicentre pragmatic randomised controlled trial with 1547 participants recruited from 24 major trauma centres across the United Kingdom between July 2016 and April 2018. The primary aim of the trial was to assess wound healing outcomes in adults who had surgical incisions for lower limb fractures related to major trauma, who were randomised to either incisional negative-pressure wound therapy or standard wound dressings. Full details of the trial sampling procedures, methodology, outcome measures and results are reported elsewhere.^10–13^ The use of trial data for these secondary analyses was permitted under the informed consent and research ethics committee approvals for the main trial.^10^ We analysed all pain and cost outcome data as a cohort study, regardless of treatment intervention as no differences were found in wound outcomes (surgical site infection/wound healing).

### Pain outcomes

Pain was measured as a secondary outcome, captured using postal questionnaires at 3 and 6 months postoperatively. Pain severity was measured using an 11-point numeric rating scale (NRS) (from 0 for ‘no pain’ to 10 for ‘pain as bad as you can imagine’). Neuropathic pain characteristics were measured using the Doleur Neuropathique Questionnaire (DN4),^14^ which was added to the trial after recruitment had commenced. The full DN4 is a 10-item clinician-administered questionnaire, but our study used the seven-item participant-reported version,^14^ which focuses on pain quality (i.e. sensory and pain descriptors). This version is validated for postal use.^14^ Participants were categorised as having one of three distinct chronic postoperative pain states (pain-free, non-neuropathic pain and neuropathic pain): those with an NRS score of zero and who were DN4 negative (defined as having a score <3) were classified as ‘pain-free’; those with an NRS score of more than zero and DN4 negative were categorised as having ‘non-neuropathic pain’ and those DN4 positive (score ≥3) were categorised as having pain with predominantly neuropathic characteristics (‘neuropathic pain’).

### Cost outcomes

Participant-reported health and social service resource use due to the trauma injury was collected using bespoke questionnaires at three- and 6-months post-surgery with a recall period at each time point of 3 months. We collected data on inpatient care after initial discharge following the lower limb fracture; hospital outpatient care (i.e. orthopaedics, pathology, radiology, physiotherapy and emergency department); community health care (i.e. general practitioner, practice nurse, district nurse, community physiotherapy, occupational therapist and calls to National Health Service (NHS) 111 or ambulance); use of personal social services (i.e. meal-on-wheels, laundry, social worker and care worker); pain medications due to the injury (i.e. NSAID, opioid, neuropathic pain medication, non-opioid analgesic, local analgesic [not NSAID] and topical NSAID); aids and adaptations; additional care (i.e. travel, child care and help with housework); as well as time off work due to the injury. Unit costs for the resource inputs within the trial were valued in UK Sterling using secondary sources and have been reported elsewhere.^15,16^ Costs are presented in 2017/18 prices and no discounting was applied as the time horizon was less than a year.

### Statistical analysis

Descriptive statistics were used to assess costs and pain medication use by pain state over two postoperative time periods (0–3 months and 4–6 months). The adjusted total mean cost over each time period for each resource use category was computed using a two-part model to account for the skewed distribution of economic costs as a result of a high frequency of people who incurred no cost and a small proportion with extremely high associated costs. The two-part model consisted of two stages, (1) a logistic regression, in which the dependent variable (total economic costs) indicated presence of zero costs (yes, no), and (2) a generalised linear model (GLM) with a gamma distribution and log link function for economic costs relating to participants with positive values. The adjusted mean total NHS and personal social services (PSS) cost over each time period was estimated using a GLM. Adjustments to the models were done using the same covariates as previous analyses,^6^ namely, allocated trial treatment (incisional negative-pressure wound therapy and standard dressing), stratification factors (injury severity score and wound closure), sex and age at randomisation. All comparisons were made against the neuropathic pain state (i.e. pain-free versus neuropathic pain and non-neuropathic pain versus neuropathic pain).

The base case analysis was completed using multiple imputed data from the NHS and PSS perspective. Multiple imputation of the missing data has been previously described^11^ but for these analyses, we have additionally assumed that if one category of resource use within a participant questionnaire was completed (e.g. community care) and if the others were not completed, values for resource use and therefore economic costs for incomplete resource categories were zero.

### Sensitivity analysis

In order to assess the robustness of the study results, two sensitivity analyses were conducted. Firstly, economic costs were computed from a societal perspective, and included direct non-medical costs such as out-of-pocket expenses, and indirect costs that valued time off work due to the injury. Secondly, a complete case analysis, in which only participants with completed data on all cost data at all follow-up time points were included, was conducted. All statistical analyses were conducted using Stata 17. A two-sided significance level of 0.05 was used throughout, and 95% confidence intervals (CIs) were reported.

## Results

Baseline characteristics of the whole WHiST cohort (*n* = 1547), and of those participants who provided postoperative pain data at three or 6 months (*n* = 934) are shown in Table 1. Of 702 participants who provided pain data at 3 months, only 84 (12%) were pain-free, 396 (56%) had non-neuropathic pain and 222 (32%) had neuropathic pain at 3 months postoperatively. By six months, 140/787 (18%) were pain-free, 413 (52%) had non-neuropathic pain and 234 (30%) had neuropathic pain. A total of 188/1547 (12%) participants reported taking regular pain medications before being randomised into the trial.

**Table 1.** Baseline characteristics of WHiST participants by postoperative pain data.

Characteristic	All participants (*n* = 1547)	Provided pain data, 3 months (*n* = 702)	Provided pain data, 6 months (*n* = 787)
Male (%)	964 (62.3)	409 (58.3)	464 (59.0)
Mean age, years (SE)	49.8 (0.5)	51.6 (0.7)	51.6 (0.7)
Race/ethnicity (%)			
White	1368 (88.4)	636 (90.6)	732 (93.0)
Black African	28 (1.8)	14 (2)	9 (1.1)
Black Caribbean	15 (1.0)	5 (0.7)	4 (0.5)
Black, other	6 (0.4)	1 (0.1)	0 (0)
Indian	18 (1.2)	11 (1.6)	11 (1.4)
Pakistani	21 (1.4)	13 (1.9)	10 (1.3)
Bangladeshi	4 (0.3)	1 (0.1)	1 (0.1)
Chinese	1 (0.1)	1 (0.1)	1 (0.1)
Other	53 (3.4)	20 (2.8)	18 (2.3)
Mean body mass index, kg/m^2^ (SE)	26.5 (0.2)	26.6 (0.2)	26.4 (0.2)
Diabetes (%)			
No	1377 (89.0)	631 (89.9)	716 (91.0)
Yes	148 (9.6)	71 (10.1)	71 (9.0)
Regular smoker (%)			
No	1068 (69.0)	525 (74.8)	607 (77.1)
Yes	434 (28.1)	177 (25.2)	179 (22.8)
Alcohol consumption per week (%)			
0–7 units	1021 (66.0)	477 (67.9)	536 (68.1)
8–14 units	215 (13.9)	102 (14.5)	117 (14.9)
15–21 units	111 (7.2)	55 (7.8)	64 (8.1)
More than 21 units	139 (9.0)	67 (9.5)	68 (8.7)
Marital status (%)			
Single	524 (33.9)	221 (31.5)	233 (29.6)
Living with a partner	210 (13.6)	85 (12.1)	107 (13.6)
Married/civil partner	524 (33.9)	270 (38.5)	318 (40.5)
Separated	37 (2.4)	19 (2.7)	20 (2.5)
Divorced	83 (5.4)	42 (6.0)	50 (6.4)
Widowed	122 (7.9)	62 (8.8)	56 (7.1)
Education/qualification (%)			
None	604 (39.0)	287 (40.9)	301 (38.2)
Formal qualification(s) through training at work	270 (17.5)	121 (17.2)	141 (17.9)
Qualification (other than a degree) from college or university	350 (22.6)	163 (23.2)	185 (23.5)
Degree from college or university	227 (14.7)	122 (17.4)	152 (19.3)
Employment status (%)			
Full-time employed	597 (38.6)	277 (39.5)	325 (41.3)
Part-time employed	109 (7.0)	50 (7.1)	62 (7.9)
Self-employed	147 (9.5)	77 (11.0)	87 (11.1)
Unemployed	172 (11.1)	68 (9.7)	65 (8.3)
Full-time student	41 (2.7)	20 (2.8)	20 (2.5)
Retired/look after home/inactive	407 (26.3)	198 (28.2)	214 (27.2)
Unpaid work	12 (0.8)	9 (1.3)	10 (1.3)
Injury severity score ≤ 15 (%)	1207 (78.0)	561 (79.9)	630 (80.2)
Mechanism of injury (%)			
Low energy fall	527 (34.1)	252 (35.9)	273 (34.7)
High energy fall	284 (18.4)	129 (18.4)	153 (19.5)
Road traffic accident	571 (36.9)	254 (36.2)	282 (35.8)
Crush injury	32 (2.1)	15 (2.1)	18 (2.3)
Contact sports	22 (1.4)	6 (0.9)	9 (1.1)
Other	103 (6.7)	46 (6.6)	52 (6.6)
Wound not closed at presentation (%)	288 (18.6)	143 (20.4)	145 (18.4)
Wound location (%)			
Femur/patella	596 (38.5)	254 (36.2)	286 (36.4)
Hip/acetabulum	325 (21.0)	141 (20.1)	166 (21.1)
Tibia/fibula/foot	619 (40.0)	307 (43.7)	335 (42.6)
Wound location, side (%)			
Left	758 (49.0)	351 (50)	383 (48.7)
Right	780 (50.4)	351 (50)	404 (51.3)
Other injuries (%)			
No	457 (29.5)	281 (40.0)	281 (35.8)
Yes	881 (56.9)	317 (45.2)	406 (51.6)
Pre-injury medication for pain (%)			
No	1334 (86.2)	628 (89.5)	705 (89.6)
Yes	188 (12.2)	74 (10.5)	82 (10.4)
Analgesia pre-injury (%)			
No	1229 (79.4)	583 (83.0)	658 (83.6)
Yes	288 (18.6)	119 (17)	128 (16.3)
Treatment allocated (%)			
Negative-pressure wound therapy	784 (50.7)	363 (51.7)	418 (53.1)
Standard dressing	763 (49.3)	339 (48.3)	369 (46.9)

**Figure 1.**
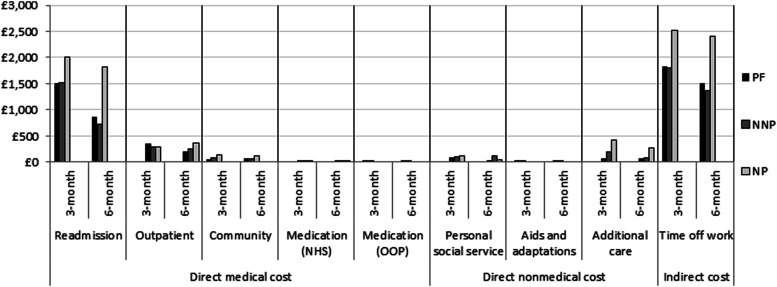
Comparison of adjusted mean cost of each resource item by pain state at three and 6 months postoperatively. NNP: Non-neuropathic pain, NP: Neuropathic pain, PF: pain-free.

Figure 1 shows the adjusted mean cost difference in resource categories by pain state at each postoperative time point. Overall, the mean cost of resource use decreased with time; with direct medical costs (i.e. readmission, outpatient care, community care and medications) constituting almost the same proportion of costs incurred as indirect costs (i.e. value of time off work) regardless of the pain states and time period of analysis. Direct non-medical costs such as PSS, aids and adaptations, as well as additional care, constituted the lowest proportion of total cost incurred. Table 2 shows that participants with chronic neuropathic pain incurred higher mean costs than those with non-neuropathic pain (3 months: £730, *p* = 0.38, 95% CI −2368 to 908; 6 months: £1,224, *p* = 0.10; 95% CI −2693 to 246) and compared to those who were pain-free (3 months: £716, *p* = 0.53, 95% CI −2929 to 1497; 6 months: £1,273, *p* =0.13; 95% CI −2938 to 393) at 3 and 6 months from the NHS and PSS perspective. Similar results were found in the sensitivity analyses where economic costs were valued from a societal perspective and where a complete case analysis was adopted.

**Table 2.** Total mean costs (£ UK Sterling) at postoperative time point by pain state.

	Randomisation to 3 months					3–6 months				
	Adjusted mean cost, £ (SE)			Adjusted mean cost difference, £ (95% CI) *p*-value		Adjusted mean cost, £ (SE)			Adjusted mean cost difference, £ (95% CI) *p*-value	
	PF	NNP	NP	PF versus NP	NNP versus NP	PF	NNP	NP	PF versus NP	NNP versus NP
(i)	*n* = 84	*n* = 396	*n* = 222			*n* = 140	*n* = 413	*n* = 234		
	2057 (769)	2043 (362)	2773 (819)	−716 (−2929 to 1497) *p* = 0.53	−730 (−2368–908) *p* = 0.38	1134 (437)	1183 (253)	2407 (736)	−1273 (−2938 to 393) *p* = 0.13	−1224 (−2693 to 246) *p* = 0.10
(ii)	*n* = 84	*n* = 396	*n* = 222			*n* = 140	*n* = 413	*n* = 234		
	4254 (1102)	4362 (523)	5738 (987)	−1484 (4443 to 1476) *p* = 0.33	−1375 (−3522 to 771) *p* = 0.21	2,39 (696)	2897 (501)	5112 (1272)	−2720 (−5494 to 54) *p* = 0.06	−2215 (−4844 to 4214) *p* = 0.10
(iii)	*n* = 84	*n* = 394	*n* = 220			*n* = 139	*n* = 412	*n* = 231		
	2046 (764)	2016 (359)	2864 (857)	−818 (−3077 to 1442) *p* = 0.48	−847 (−2546 to 851) *p* = 0.33	1158 (444)	1181 (251)	2460 (751)	−1302 (−3002 to 397) *p* = 0.13	−1279 (−2776 to 219) *p* = 0.10

NNP: non-neuropathic pain, NP: neuropathic pain, PF: pain-free, SE: standard error.

(i) Base case analysis from the NHS and Personal Social Services (PSS) perspective; (ii) Sensitivity analysis from the societal perspective; (iii) Sensitivity analysis: complete case from the NHS and PSS perspective.

*p*-value was computed using generalised linear model.

Data on pain medication usage by prescription type, postoperative time point and pain state is summarised in Table 3. Opioids were the most frequently reported prescribed medications consumed at 3 (155/702; 22%) and 6 (85/787; 11%) months.

**Table 3.** Number (%) taking prescribed versus over-the-counter pain medications at postoperative time point by pain states.

Time point	Type of prescription	Type of pain medication^a^	PF	NNP	NP
Randomisation to 3 months			*n* = 84	*n* = 396	*n* = 222
	Prescribed	NSAID	0 (0.0)	10 (2.5)	18 (8.1)
		Non-opioid analgesic	1 (1.2)	30 (7.6)	24 (10.8)
		Opioid	9 (10.7)	81 (20.5)	65 (29.3)
		Neuropathic pain	1 (1.2)	11 (2.8)	17 (7.7)
		Local analgesic	0 (0.0)	0 (0.0)	0 (0.0)
		Topical NSAID	0 (0.0)	1 (0.3)	1 (0.5)
	Over-the-counter	NSAID	1 (1.2)	12 (3.0)	12 (5.4)
		Non-opioid analgesic	3 (3.6)	32 (8.1)	26 (11.7)
		Opioid	0 (0.0)	4 (1.0)	2 (0.9)
		Neuropathic pain	0 (0.0)	0 (0.0)	0 (0.0)
		Local analgesic	0 (0.0)	0 (0.0)	0 (0.0)
		Topical NSAID	0 (0.0)	1 (0.3)	0 (0.0)
3–6 months			*n* = 140	*n* = 413	*n* = 234
	Prescribed	NSAID	2 (1.4)	9 (2.2)	7 (3.0)
		Non-opioid analgesic	2 (1.4)	10 (2.4)	17 (7.3)
		Opioid	4 (2.9)	33 (8.0)	48 (20.5)
		Neuropathic pain	2 (1.4)	13 (3.1)	13 (5.6)
		Local analgesic	0 (0.0)	0 (0.0)	2 (0.9)
		Topical NSAID	0 (0.0)	0 (0.0)	0 (0.0)
	Over-the-counter	NSAID	0 (0.0)	11 (2.7)	9 (3.8)
		Non-opioid analgesic	1 (0.7)	18 (4.4)	21 (9.0)
		Opioid	0 (0.0)	0 (0.0)	3 (1.3)
		Neuropathic pain	0 (0.0)	0 (0.0)	0 (0.0)
Local analgesic		0 (0.0)	0 (0.0)	0 (0.0)	
Topical NSAID		0 (0.0)	1 (0.2)	0 (0.0)	

NNP: non-neuropathic pain, NP: Neuropathic pain, PF: Pain-free.

^a^Breakdown of each type of pain medication: (1) Non-steroidal anti-inflammatory drug (NSAID) are diclofenac, ibuprofen and naproxen; (2) non-opioid analgesic are co-proxamol and paracetamol; (3) opioid are buprenorphine, co-codamol, codeine, co-dydramol, dihydrocodeine, fentanyl, meptazinol, methadone, morphine, oxycodone, solpadeine, tramadol and zomorph; (4) neuropathic pain are amitriptyline, duloxetine, gabapentin and pregabalin; (5) local analgesic are algesal cream and lidocaine and (6) topical NSAID is voltarol.

During the first 3-month period, one-third of participants (65/222, 30%) with neuropathic pain were prescribed opioids, compared to one-fifth (81/396, 21%) of those with non-neuropathic pain. The proportion of participants prescribed opioids decreased between 3 and 6 months, with 48/234 (21%) of those with neuropathic pain and 33/413 (8%) with non-neuropathic pain prescribed opioids by 6 months. Non-opioid analgesics were the most common over-the-counter medications purchased; 26/222 (12%) and 21/234 (9%) with neuropathic pain bought non-opioid analgesics over-the-counter at 3 and 6 months, respectively. Likewise, those with non-neuropathic pain (32/396, 8% and 18/413, 4%) bought non-opioid analgesics over-the-counter at 3 and 6 months, respectively. During the first 3 months after injury, only 17/222 (8%) of participants were prescribed medications specifically indicated for neuropathic pain management; this proportion also remained low at 6 months (13/234, 6%).

## Discussion and conclusions

This study found that among people with surgically treated lower limb fractures following major trauma, those experiencing chronic pain with neuropathic characteristics over the first six postoperative months incurred higher costs from a UK NHS and PSS perspective as well as from a societal perspective than those who were pain-free or had non-neuropathic pain. This is consistent with several other studies that investigated the association between neuropathic pain and economic outcomes in trauma patients.^8,17^ The European burden of illness study conducted by Liedgens et al.^5^ showed that indirect costs (€5,492, in 2012 prices) were approximately twice the amount of direct medical costs (€2,951, in 2012 prices) amongst patients with neuropathic pain seeking treatment while the American study by Schaefer et al.^8^ concluded that indirect cost was the main cost driver of costs associated with neuropathic pain. Unlike the aforementioned studies, we found that direct medical costs constituted almost the same amount as indirect costs across the different pain states.

Our detailed data on medication use collected as part of health resource use data collection raises important questions about medication usage in participants with surgically treated lower limb fractures. In the first 3 months after injury, opioids were used by about one in five overall. The continued use of opioids between three and six postoperative months by 11% of the whole cohort, and by approximately one in five people with neuropathic pain is notable. The UK NICE guidelines on chronic pain do not recommend opioids for chronic pain management.^7^ The related NICE guidance for pharmacological management of neuropathic pain includes recommendations to consider amitriptyline, duloxetine, gabapentin or pregabalin as the first line of pharmacological therapy.^9^ Our medication use data indicate that clinical practice contrasted with these recommendations, with only about 1 in 13 participants and 1 in 15 participants experiencing chronic neuropathic pain using these medications over each time period, respectively. These findings highlight potential inappropriate pharmacological management. The use of opioids at the rate observed in this cohort could have serious implications given the data from other trauma cohort studies showing the adverse effects of opioid use. For example, a Swedish study including 13,309 injured patients and 70,621 uninjured matched controls found that trauma was independently associated with long-term opioid use (odds ratio 3.28, 95% CI 3.02–3.55), and long-term opioid was associated with an increased risk of all-cause mortality at 6–18 months post-injury (hazard ratio 1.82, 95% CI 1.34–2.48).^18^ The potential under-treatment of neuropathic pain from our data also highlights that this type of pain presentation may not be being consistently identified and treated in this patient group. These findings have implications for clinical education regarding pain assessment and management, and certainly highlight the need for further research to explore how best to undertake pain assessment, prescribe appropriate pharmacological therapies and supportive strategies after major lower limb injuries.

A key strength of this study lies in the comprehensive high-quality data collection as the WHiST trial is one of the largest cohorts of major trauma patients in the United Kingdom to date, with neuropathic pain data captured over the first 6 months after surgery. The resource use data allowed the capture of healthcare activity that would otherwise not have been available from routine datasets or registry data and thus enabled the estimation of a broad spectrum of economic costs, including direct non-medical costs and indirect costs. A key limitation is the use of a self-reported neuropathic screening questionnaire, without detailed clinical assessment to further establish a confirmatory diagnosis of neuropathic pain.^19^ However, as the DN4 self-report has excellent sensitivity (78%) and specificity (81%) compared to the longer version with clinical assessment,^14^ and given that this was a large population-wide cohort, the self-report questionnaire was feasible and appropriate in our context. Furthermore, as this is the secondary analysis of a trial dataset, the original sample size calculation was not based on these research questions and because the DN4 was added to the trial after recruitment had commenced, so it is likely that we did not have enough power for our analyses to reach statistical significance. Nevertheless, the clinical relevance of these findings is timely and important for the orthopaedic and pain community.

In conclusion, our study found that amongst people with surgical wounds from lower limb fractures that could be primarily closed following major trauma, those with chronic neuropathic pain incurred higher NHS and PSS costs and societal costs compared to those who were pain-free or had chronic non-neuropathic pain. Pain medication usage, particularly opioid consumption was common although neuropathic pain medications were infrequently prescribed, contrary to clinical guidelines on persistent neuropathic pain management.
